# Cochlear Implantation in Children Affected by Single-Sided Deafness: A Comprehensive Review

**DOI:** 10.3390/audiolres14010007

**Published:** 2024-01-12

**Authors:** Giuseppe Santopietro, Virginia Fancello, Giuseppe Fancello, Chiara Bianchini, Stefano Pelucchi, Andrea Ciorba

**Affiliations:** 1 ENT & Audiology Unit, Department of Neurosciences, University Hospital of Ferrara, 44121 Ferrara, Italy; 2 Department of Otorhinolaryngology, Careggi University Hospital, 50134 Florence, Italy

**Keywords:** children, single-sided deafness, cochlear implants

## Abstract

Children with single-sided deafness (SSD) may experience delays in language and speech development. Reduced speech discrimination and poor sound localization abilities in young SSD patients may result in greater cognitive efforts required to focus and process auditory information, as well as increased listening-related fatigue. Consequently, these children can have a higher risk of academic failure and are often in need of extra help at school. Recently, cochlear implants (CIs) have been introduced as a rehabilitative option for these children, but their effectiveness is still a topic of debate. A literature review was performed according to PRISMA guidelines, searching the Medline database from inception to October 2023. The research identified nine papers that met the inclusion criteria. Data extracted from the selected studies included 311 children affected by SSD and cochlear implants. The reported audiological outcomes were further analyzed. Overall, a high level of satisfaction was described by parents of children with SSD and CI, and those who received a CI under the age of 3 presented better results. However, a proportion of patients did not use the device daily. Our review highlights the possible, and still controversial, role of CI for the hearing rehabilitation of children with unilateral deafness, underlining the need for further research in this field. To date, careful and comprehensive counseling with the child and the family is necessary before considering this option.

## 1. Introduction 

Unilateral deafness, also known as single-sided deafness (SSD), is a condition characterized by severe-to-profound hearing thresholds (pure-tone average, PTA, >70 dB) in one ear and a normal hearing threshold (PTA ≤ 25 dB) assessed by pure-tone audiometry in the contralateral [1]. The reported incidence of congenital SSD is 1:1000 births, while the prevalence of congenital and acquired SSD in pediatric patients aged between 6 and 19 years old is approximately 14% [2]. Among adults, SSD prevalence is estimated at about 0.14% [1].

The impact of hearing loss on global health is becoming further recognized, but the disabilities related to SSD are often underestimated. Binaural hearing is compromised by profound unilateral hearing loss, reducing an individual’s ability to localize sounds and process speech in a noisy environment, with consequent auditory and social implications, and an overall deterioration of life quality perception [3].

According to the literature, children with unilateral hearing loss can have delays in language and speech development. Reduced speech discrimination and poor sound localization abilities in young SSD patients may result in greater cognitive efforts required to focus and to process auditory information, as well as increased listening-related fatigue. Consequently, these children can have a higher risk of academic failure and are often in need of further help at school, as reported by Fischer and Lieu [4,5]. In addition, compared to their peers, they have also been found to score lower intelligence quotients (IQs) [4,5].

In the past, bone-conducted implants, or contralateral routing of the signal (CROS) hearing aids, were the primary options for SSD rehabilitation. However, these devices have been reported to improve only partially sound localization or speech understanding in a competitive environment and cannot truly restore binaural hearing [6,7,8]. 

Firstly, developed in 1961, cochlear implantation (CI) is nowadays the only effective treatment to restore useful hearing in profound deafness and could represent a possible choice for individuals with profound unilateral hearing loss in order to reestablish binaural hearing [9]. In fact, CIs have been reported to improve speech understanding in quiet and noise, sound localization, and therefore quality of life perception in those affected by SSD [9]. This interest in restoring binaural inputs to patients with SSD with cochlear implants has grown during the last decade [3,10], and several studies reporting improvements in speech recognition in noise, sound localization, and tinnitus control have been published.

SSD differs greatly from other types of sensorineural hearing loss, and ENT and healthcare professionals must carefully evaluate CI as a rehabilitative option for these patients.

While for adults with SSD, the use of CI is an acceptable and beneficial hearing rehabilitation option, for children with unilateral hearing loss, there are not clear guidelines yet. In fact, only a few studies have focused on the outcomes of cochlear implantation in children with SSD. 

The aim of this paper is to assess the clinical and audiological outcomes in children affected by SSD and treated by CI through a literature review. 

## 2. Materials and Methods

A literature search of English-language studies on the use of CI in SSD pediatric patients was performed using the Medline database. The mesh terms “unilateral hearing loss” and “Cochlear Implant” were used in combination with the additional filter for children aged 0–18 years. The query resulted in 66 candidate papers to which the following criteria were applied.

Inclusion criteria:

- Original studies on cohorts > 10 patients (in order to identify studies with an adequate sample size [6]);
- Studies on young subjects, defined as those aged 18 years and under (https://www.who.int/health-topics/ageing#tab=tab_1, accessed on 30 November 2023);
- Studies including patients affected by SSD according to the definition of SSD by the American Audiology Academy.

Exclusion criteria:

- Studies containing duplicated data from other published work;
- Cohort of patients < 10;
- Studies published in a non-English language;
- Studies not including audio-vestibular diagnoses;
- Studies analyzing only specific subgroups of diagnoses;
- Reviews, letters, and case reports.

**Figure 1:**
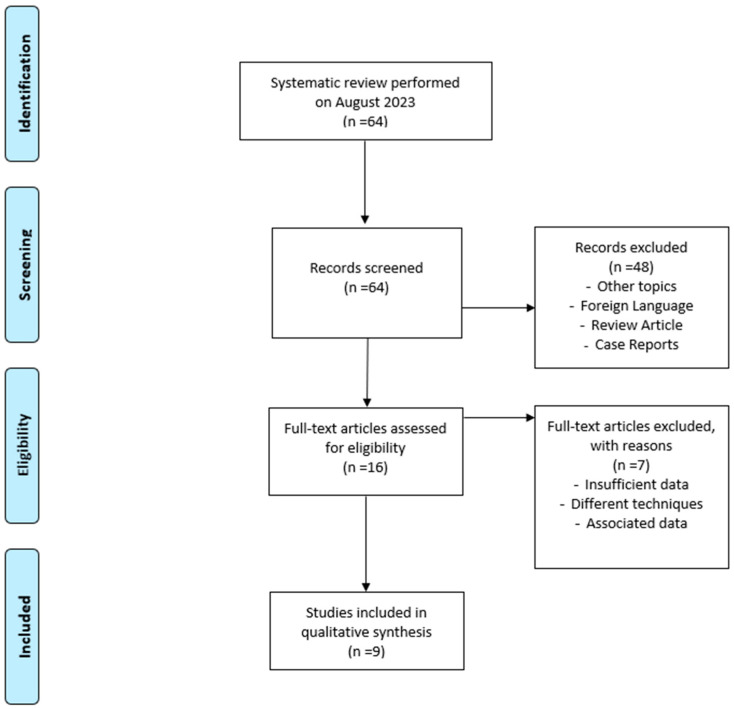
Prisma flow diagram.

Of the initial candidates, only 9 papers met the inclusion criteria, of which 1 article was used exclusively for the analysis of the etiology of SSD due to the limited information on the CI used by patients. The review was conducted using the Preferred Reporting Items for Systematic Reviews and Meta-Analyses (PRISMA) guidelines. The flow diagram is illustrated in Figure 1. 

## 3. Results

Upon the application of the above-mentioned criteria, we selected nine studies for further analysis (Table 1). The total population included 311 patients. 

Three papers did not provide information on patient sex [13,15,17], while in the remaining, 52% were male and 48% female, with a male-to-female ratio of 1:1.1.

The mean age was 6 years old, ranging from 6 months to 17 years old.

The type of research included five retrospective studies, three prospective studies, and one observational study. 

These nine papers were published from 2017 to 2023 in different countries (Canada, USA, Germany, Spain, and Israel). 

**Figure 2:**
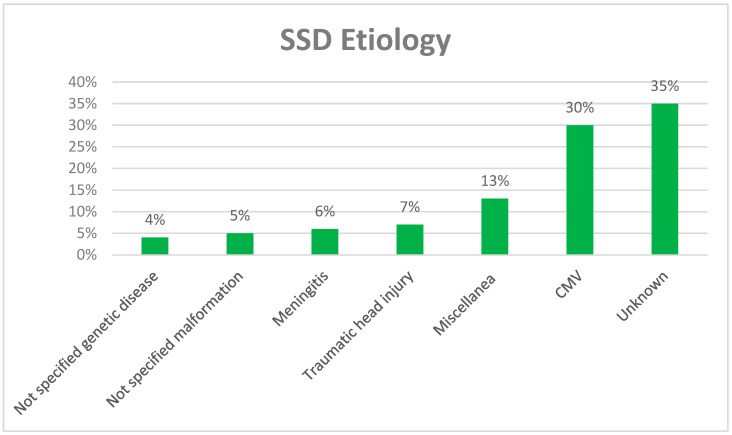
Etiology of SSD in the paper selected for review.

The most frequent cause of SSD in children was cytomegalovirus (CMV), which accounted for 30% of cases, followed by traumatic head injury (7%) and meningitis (6%) (Figure 2). The miscellaneous category, which accounts for 5%, includes idiopathic sudden hearing loss, enlarged vestibular aqueduct, perinatal hypoxia, ototoxicity, Waardenburg syndrome, cholesteatoma, Langerhans’ histiocytosis, cochlear nerve aplasia, dysplasia, hypoplasia, and cochlear incomplete partition. In 35% of SSD cases, the etiology remains unknown. 

The length of hearing deprivation was reported in all except one study, ranging from 2 to 14 years [11,12,14,16,17,19]. Further details of the auditory features of the studied cohorts are illustrated in Table 2.

CI manufacturers were Advanced Bionics in 1.6%, Cochlear in 45.6%, and Med-EL in 37.2% of cases; in the remaining 15.6% of cases, the details of the CI brand were not specified.

**Figure 3:**
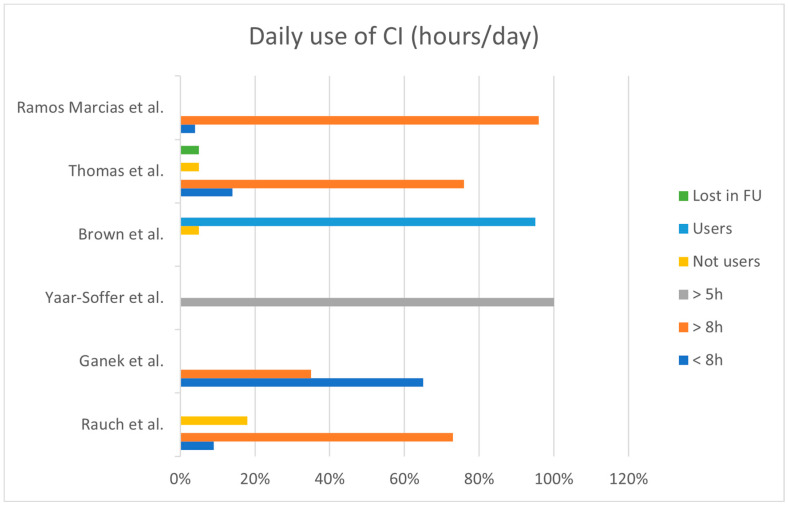
Overall use of CI (hours/day) within the selected studies. (h = hours; FU = follow-up) [11,12,14,15,16,18].

CI use (hours per day) of patients was reported only in four studies [11,12,14,15] that divided the users into two major subgroups: those using CI more than 8 h per day and those using CI less than 8 h (Figure 3). Overall, the reported use of CI is more than 8 h per day in 70% of the cases, and less than 8 h per day in 26%; three children (4%) have been reported to be non-users.

One study [18] specified that all children used their CI more than 5 h per day, while Brown et al. reported that 95% of their patients used the CI daily, and one patient was a suspected non-user.

Self-administrated questionnaires, designed for both children and their parents, in order to assess family satisfaction after CI and quality of life, have been administered by six out of nine studies [11,12,15,16,17,18]. The tools used were the Speech, Spatial, and Qualities of Hearing Scale (SSQ) [20], the International Outcome Inventory for Hearing Aids (IOI-HA) [21], and the Pediatric Quality of Life Inventory (PedsQL) [13].

Overall, a high level of satisfaction was described by parents of implanted SSD children, and those who received a CI under the age of 3 presented better results [15]. According to the SSQ questionnaire, better benefits were reported in terms of hearing and speech quality than spatial hearing. 

## 4. Discussion 

Individuals with single-sided deafness (SSD) face many challenges that can profoundly affect their quality of life. In particular, the loss of binaural hearing compromises sound localization [22] and the comprehension of speech in noisy environments, leading to communication barriers. Moreover, those with SSD struggle with immediate hearing impediments caused by the “head shadow effect”. They also encounter limitations related to the “squelch effect”, which reduces the advantages of incorporating the ear with a weaker signal-to-noise ratio, and the “summation effect”, which amplifies perceived loudness when both ears detect a sound signal. All these features can affect different aspects of their hearing and of their daily lives, as they can interfere not only with daily activities but also with learning and educational acquisitions. 

The impact of SSD extends beyond age groups, and, furthermore, there is substantial evidence supporting the long-term effects of SSD. Among pediatric patients, SSD is recognized for its impact on speech, language, and eventually on academic performances, as highlighted by some authors [19]. These challenges can persist into adulthood, causing adverse consequences for social interactions, learning capacities, and occupational performance. These eventual negative consequences for educational and professional achievements can also result in reduced opportunities and decreased quality-of-life perception [23].

CROS hearing aids and bone-conduction hearing devices have been reported for the treatment of SSD [1]. However, these means are limited in their ability to restore true binaural hearing since they just route auditory inputs to the normal hearing ear. In particular, their efficacy in terms of improving sound localization and speech perception in noisy environments has been reported to be limited [4]. 

On the contrary, CIs could offer encouraging options for the treatment of SSD. Some researchers have indicated that the central neural adaptations following CI in SSD patients may differ from those with bilateral hearing loss, potentially enabling the integration of electric and acoustic signals [6]. Although the full extent of this signal integration is still under investigation, it supports the subjective evidence of enhanced hearing capabilities [24].

According to the data of the current review, it is likely that children affected by SSD may benefit more from CI when a cochlear implant is placed early (<3 years old) [11], possibly due to a further maturation of the auditory pathway and myelinization processes that have been reported to begin before birth and then continue up to the 4th year of life [6,25]. However, data on these features are still inconsistent. Ardt et al. [26] described a better performance in children with post-verbal unilateral hearing loss than in children with pre-verbal or congenital SSD, who had worse outcomes in verbal discrimination and auditory localization. Instead, Rahne and Plontke [27] demonstrated satisfactory results using CI in pre-verbal and congenital SSD. The central brain adaptations, occurring post CI in SSD, differ from those in subjects affected by bilateral hearing loss, and it is still unknown how patients with SSD and those rehabilitated by CI can integrate electric and physiological acoustic stimulation over time [28]. Probst hypothesized the lack of development of central compensation mechanisms in children with SSD and then cochlear implanted [29]; this fact, together with the above-mentioned considerations, makes the choice of CI rehabilitation in SSD children very complex. Yaar-Soffer Y et al. have evaluated the neuro-plasticity of the central auditory pathways after CI through the use of cortical auditory evoked potentials, demonstrating the improved abilities of these children [18]. In particular, the available data corroborate the beneficial benefits of electrical stimulation, including improved myelination and expanded neuronal connections within the auditory pathway. Furthermore, Sharma et al. demonstrated, using electroencephalography data, that even after several years of unilateral hearing deprivation, children can eventually present neuro-plasticity patterns within the auditory cortex after cochlear implantation [30]. Therefore, late implantation could eventually enhance the processing of auditory information in the brain. In our opinion, further studies are necessary in this field.

Concerning the possible effects of CI on tinnitus, there are still not enough data available in the pediatric population [30,31,32,33]; therefore, it is not possible to draw a firm conclusion on this specific topic.

Counseling with the family is a decisive step before the CI indication in SSD, particularly since it has been shown that a certain proportion of these children became non-users. Aside from the neurophysiological reasons, children can become non-users due to other causes, such as a lack of family support or emotional distress [31]. The main feature linked to CI efficiency is represented by its daily use. Despite the fact that a large proportion of children included in this review have been reported to use CI for more than 8 h per day, a negligible portion of subjects were classified as non-users. Since the best CI performance can only be achieved through relevant educational and familiar support [32], the use of the CI may be negatively affected by a lack of this support, particularly if family expectations about CI outcomes are not fulfilled. Another factor that can eventually limit CI use (in terms of hours per day) is that children might experience subjective benefits from the device only in specific circumstances, such as school. 

Furthermore, during adolescence, the presence of the device may attract negative attention [33], particularly due to the presence of the external processor. This fact, combined with a possible mismatch between teenager expectations and the reality of CI performance, may also lead the teenager to abandon the device.

Finally, the etiology of SSD may also eventually influence the performance and use of the device. It has been reported that patients with SSD secondary to congenital CMV could have worse performance and variable results compared to their implanted peers. Also, cochlear nerve hypoplasia is reported in a variable percentage of children with SSD [34], and those affected are less likely to respond to the electrical stimulus generated by the CI. MRI is always essential to identify this condition and to avoid erroneous indications and management [27].

## 5. Conclusions

This review paper highlights the possible, and still controversial, role of CI in improving the quality of life of children with unilateral deafness. Cochlear implants can restore hearing and improve sound localization abilities, reducing auditory fatigue and therefore increasing concentration and scholar performances. In adults, CIs are also known to effectively manage tinnitus and reduce vertigo symptoms, and it is likely that these effects should be further explored in pediatric patients as well. 

The outcomes of the present review emphasize the need for further research and development in this field, which can possibly ameliorate the quality of life of SSD subjects by improving their social and educational skills and eventually their career opportunities. Overall, CI can represent a treatment option for children with SSD by improving not only hearing but also overall well-being; however, to date, careful and comprehensive counseling with the child and the family is necessary before considering this option. 

## Figures and Tables

**Table 1:** Characteristics of the studies (Ref. = references; P = prospective study; R = retrospective study; O = observational study; # = number of patients; M = male; F = female; y. = years; m. = months).

Authors	Ref.	Year	Country	#	Sex	Age	Study
Thomas et al.	[11]	2017	Germany	21	M: 8, F: 13	9 m.–11 y.	R
Ramos Marcias et al.	[12]	2018	Spain	23	M: 11, F: 12	11 m.–11 y.	O
Cushing et al.	[13]	2019	Canada	37	-	-	R
Ganek et al.	[14]	2019	Canada	23	M: 13, F: 10	1–15 y.	R
Rauch et al.	[15]	2020	Germany	11	-	1–13 y.	R
Brown et al.	[16]	2022	USA	20	M: 12, F: 8	3–12 y.	P
Gordon et al.	[17]	2023	Canada	57	-	1–15 y.	P
Yaar-Soffer et al.	[18]	2023	Israel	22	M: 16, F: 6	1–8 y.	R
Park et al.	[19]	2023	USA	97	M: 47, F: 50	6 m.–17 y.	P
Summary	-	2017–2023	-	311	F:M = 1:1.1	6 m.–17 y.	-

**Table 2:** Available auditory features of the implanted SSD children within the selected studies (PTA = pure tone average, y = years, m = months, # = number of patients).

Authors	Pre-Verbal	Post Verbal	PTA Normal Ear	PTA Deaf Ear	Deprivation (Range)	Use of Hearing Aid Pre-CI (#)
Thomas et al. [11]	21	0	-	-	6 y	-
Ramos Marcias et al. [12]	4	19	-	-	1 y 3 m	-
Cushing et al. [13]	-	-	20	100	<4 y	-
Ganek et al. [14]	13	10	<25	>90	2 y	-
Rauch et al. [15]	9	2	<20	>90	From 1 y to 13 y	-
Brown et al. [16]	11	9	10	108	3 y 3 m	6
Gordon et al. [17]	40	17	<25	-	1 y 9 m	-
Yaar-Soffer et al. [18]	20	2	<20	>80	-	-
Park et al. [19]	53	44	-	-	From 2 m to 14 y	-
Summary	171	103	<25	>80		

## Data Availability

The data collected in this study are available from the corresponding author upon reasonable request.

## References

[1] Kay-Rivest E., Irace A.L., Golub J.S., Svirsky M.A. (2022). Prevalence of Single-Sided Deafness in the United States. Laryngoscope.

[2] Lieu J.E. (2018). Permanent unilateral hearing loss (UHL) and childhood development. Curr. Otorhinolaryngol. Rep..

[3] Caruso A., Giannuzzi A.L., Sozzi V., Sanna M. (2017). Bone anchored hearing implants without skin thinning: The Gruppo Otologico surgical and audiological experience. Eur. Arch. Otorhinolaryngol..

[4] Lieu J.E. (2004). Speech-language and educational consequences of unilateral hearing loss in children. Arch. Otolaryngol. Head Neck Surg..

[5] Fischer C., Lieu J. (2014). Unilateral hearing loss is associated with a negative effect on language scores in adolescents. Int. J. Pediatr. Otorhinolaryngol..

[6] Hansen M.R., Gantz B.J., Dunn C. (2013). Outcomes after cochlear implantation for patients with single-sided deafness, including those with recalcitrant Meniere’s disease. Otol. Neurotol..

[7] Buss E., Dillon M.T., Rooth M.A., King E.R., Deres E.J., Buchman C.A., Pillsbury H.C., Brown K.D. (2018). Effects of cochlear implantation on binaural hearing in adults with unilateral hearing loss. Trends Hear..

[8] Peters J.P.M., Smit A.L., Stegeman I., Grolman W. (2015). Review: Bone conduction devices and contralateral routing of sound systems in single-sided deafness. Laryngoscope.

[9] Mertens G., De Bodt M., Van de Heyning P. (2017). Evaluation of Long-Term Cochlear Implant Use in Subjects with Acquired Unilateral Profound Hearing Loss: Focus on Binaural Auditory Outcomes. Ear Hear..

[10] Arndt S., Aschendorff A., Laszig R., Beck R., Schild C., Kroeger S., Ihorst G., Wesarg T. (2011). Comparison of pseudobinaural hearing to real binaural hearing rehabilitation after cochlear implantation in patients with unilateral deafness and tinnitus. Otol. Neurotol..

[11] Thomas J.P., Neumann K., Dazert S., Voelter C. (2017). Cochlear Implantation in Children with Congenital Single-Sided Deafness. Otol. Neurotol..

[12] Ramos Macías Á., Borkoski-Barreiro S.A., Falcón González J.C., de Miguel Martínez I., Ramos de Miguel Á. (2019). Single-sided deafness and cochlear implantation in congenital and acquired hearing loss in children. Clin. Otolaryngol..

[13] Cushing S.L., Gordon K.A., Sokolov M., Papaioannou V., Polonenko M., Papsin B.C. (2019). Etiology and therapy indication for cochlear implantation in children with single-sided deafness: Retrospective analysis. HNO.

[14] Ganek H.V., Cushing S.L., Papsin B.C., Gordon K.A. (2020). Cochlear Implant Use Remains Consistent Over Time in Children with Single-Sided Deafness. Ear Hear..

[15] Rauch A.K., Arndt S., Aschendorff A., Beck R., Speck I., Ketterer M.C., Jakob T.F., Hassepass F. (2021). Long-term results of cochlear implantation in children with congenital single-sided deafness. Eur. Arch. Otorhinolaryngol..

[16] Brown K.D., Dillon M.T., Park L.R. (2022). Benefits of Cochlear Implantation in Childhood Unilateral Hearing Loss (CUHL Trial). Laryngoscope.

[17] Gordon K.A., Alemu R., Papsin B.C., Negandhi J., Cushing S.L. (2023). Effects of Age at Implantation on Outcomes of Cochlear Implantation in Children with Short Durations of Single-Sided Deafness. Otol. Neurotol..

[18] Yaar-Soffer Y., Kaplan-Neeman R., Greenbom T., Habiballah S., Shapira Y., Henkin Y. (2023). A cortical biomarker of audibility and processing efficacy in children with single-sided deafness using a cochlear implant. Sci. Rep..

[19] Park L.R., Gagnon E.B., Dillon M.T. (2023). Factors that influence outcomes and device use for pediatric cochlear implant recipients with unilateral hearing loss. Front. Hum. Neurosci..

[20] Gatehouse S., Noble W. (2004). The speech, spatial and qualities of hearing scale (SSQ). Int. J. Audiol..

[21] Cox R.M., Alexander G.C. (2002). The International Outcome Inventory for Hearing Aids (IOI-HA): Psychometric properties of the English version. Int. J. Audiol..

[22] Arras T., Boudewyns A., Swinnen F., Zarowski A., Philips B., Desloovere C., Wouters J., van Wieringen A. (2022). Longitudinal auditory data of children with prelingual single-sided deafness managed with early cochlear implantation. Sci. Rep..

[23] Benchetrit L., Ronner E.A., Anne S., Cohen M.S. (2021). Cochlear Implantation in Children with Single-Sided Deafness: A Systematic Review and Meta-analysis. JAMA Otolaryngol. Head Neck Surg..

[24] Vashishth A., Fulcheri A., Prasad S.C., Dandinarasaiah M., Caruso A., Sanna M. (2018). Cochlear Implantation in Chronic Otitis Media with Cholesteatoma and Open Cavities: Long-term Surgical Outcomes. Otol. Neurotol..

[25] Kinney H.C., Brody B.A., Kloman A.S., Gilles F.H. (1988). Sequence of central nervous system myelination in human infancy. II. Patterns of myelination in autopsied infants. J. Neuropathol. Exp. Neurol..

[26] Arndt S., Prosse S., Laszig R., Wesarg T., Aschendorff A., Hassepass F. (2015). Cochlear implantation in children with single-sided deafness: Does aetiology and duration of deafness matter?. Audiol. Neurootol..

[27] Rahne T., Plontke S.K. (2016). Functional Result After Cochlear Implantation in Children and Adults with Single-sided Deafness. Otol. Neurotol..

[28] Sullivan C.B., Al-Qurayshi Z., Zhu V., Liu A., Dunn C., Gantz B.J., Hansen M.R. (2020). Long-term audiologic outcomes after cochlear implantation for single-sided deafness. Laryngoscope.

[29] Probst R. (2008). Kochleaimplantation bei einseitiger Taubheit? Cochlear implantation for unilateral deafness?. HNO.

[30] Sharma A., Glick H., Campbell J., Torres J., Dorman M., Zeitler D.M. (2016). Cortical Plasticity and Reorganization in Pediatric Single-sided Deafness Pre- and Postcochlear Implantation: A Case Study. Otol. Neurotol..

[31] Deep N.L., Gordon S.A., Shapiro W.H., Waltzman S.B., Roland J.T., Friedmann D.R. (2021). Cochlear Implantation in Children with Single-Sided Deafness. Laryngoscope.

[32] Holt R.F., Beer J., Kronenberger W.G., Pisoni D.B., Lalonde K. (2012). Contribution of family environment to pediatric cochlear implant users’ speech and language outcomes: Some preliminary findings. J. Speech Lang Hear Res..

[33] Watson V., Verschuur C., Lathlean J. (2016). Exploring the experiences of teenagers with cochlear implants. Cochlear Implants Int..

[34] Ward K.M., Coughran A.J., Lee M., Fitzgerald M.B., Cheng A.G., Chang K.W., Ahmad I.N. (2023). Prevalence of Cochlear Nerve Deficiency and Hearing Device Use in Children with Single-Sided Deafness. Otolaryngol. Head Neck Surg..

